# Engineering of the Thiamine Diphosphate‐Dependent JanthE for the Synthesis of Tertiary Alcohols

**DOI:** 10.1002/chem.202500890

**Published:** 2025-06-08

**Authors:** Lucrezia Lanza, Daniela Bjarnesen, Mehmet Mervan Çakar, Andrea Rizzo, Akash Pandya, Carmen Aranda, Zvjezdana Findrik Blažević, Michael Müller

**Affiliations:** ^1^ Institute of Pharmaceutical Sciences Albert‐Ludwigs‐Universität Freiburg Albertstrasse 25 Freiburg 79104 Germany; ^2^ Faculty of Chemical Engineering and Technology University of Zagreb Trg Marka Marulića 19 Zagreb 10000 Croatia; ^3^ Johnson Matthey 28 Cambridge Science Park Milton Road Cambridge CB4 0FP UK

**Keywords:** α‐hydroxy ketones, biocatalysis, carboligation, protein engineering, ThDP

## Abstract

The development of (bio)catalytic methods to chiral tertiary alcohols is in demand, yet the enantioselective synthesis of tertiary alcohols with sterically hindered moieties is problematic. JanthE is a novel thiamine diphosphate‐dependent enzyme that catalyzes aldehyde‐ketone cross‐coupling reactions with a wide range of donor and acceptor substrates. However, product formation is low and precludes large‐scale production. We investigated the conversion of 2‐oxobutanoate and the bulky ketone phenoxy‐2‐propanone to the product 2‐hydroxy‐2‐methyl‐1‐phenoxypentan‐3‐one as a model reaction for the synthetic capabilities of JanthE. As the reaction design did not significantly improve the yield, we proceeded to rational protein engineering. Docking experiments identified V121, Y268, P293, Y297, and K567 in the active site as important residues for catalysis. Remarkably, the single‐point variant JanthE K567S led to a fivefold increase in product formation (90% *ee*) compared to the wild type enzyme (93% *ee*). The reaction was performed at preparative scale proving the direct possibility of application. Additionally, when the reaction was extended to longer 2‐oxopentanoate, the double variant Y297P_K567S showed a 5‐fold increase in product formation compared to the wild type. Our results demonstrate the evolvability and versatility of JanthE for the enantioselective synthesis of sterically hindered tertiary alcohols.

## Graphical abstract

The synthesis of chiral tertiary alcohols is in high demand. Here we investigate in detail the aldehyde–ketone cross‐coupling reactions catalyzed by the promising thiamine diphosphate‐dependent lyase JanthE. Focusing on both reaction parameters and engineering, we rationally develop JanthE to improve its applicability and demonstrate its evolvability and versatility for the enantioselective synthesis of sterically hindered tertiary alcohols.

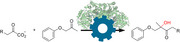

## 1 Introduction

**Figure 1:**
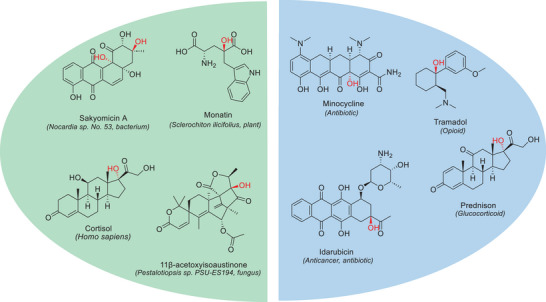
Common tertiary alcohol motifs found in natural products (left). Examples of tertiary alcohol motifs found in pharmaceuticals (right).

Chiral tertiary alcohols are ubiquitous moieties of natural products and serve as important building blocks for biologically active compounds and pharmaceuticals in the fine‐chemical and pharmaceutical industries (Figure 1). While the demand for chiral tertiary alcohols is high, the asymmetric synthesis of this essential structural moiety remains challenging. Conventional organic synthesis approaches,^[^ ^1^ ^]^ including the well‐known addition of an organometallic reagent to a ketone,^[^ ^2^, ^3^ ^]^ are limited by background reactions, enantioselectivity, toxicity issues, and lengthy purification methods.^[^ ^4^, ^5^, ^6^ ^]^ Therefore, research has focused on exploring more efficient and biocatalytic strategies. Nature is an inspiring source of diverse and versatile structures as well as the biochemical means to access these intriguing chemical motifs through enzymes. Epoxide hydrolases can be used for the ring‐opening of quaternary epoxides, while a few lipases and esterases are available for the racemic resolution of tertiary esters by hydrolysis.^[^ ^7^, ^8^, ^9^ ^]^ The sterically demanding substrates, low substrate promiscuity, and low yields are some of the challenges that still need to be overcome for their use in biocatalytic processes. P450 monooxygenases can catalyze the asymmetric hydroxylation of tertiary carbon substrates at C–H bonds. Although recent progress has been made in this field,^[^ ^10^ ^]^ the selective activation of such sterically hindered positions is not easy to accomplish.

Enzymatic processes in which C–C bonds are formed are less common, but advantageous compared to racemic resolution, as they give a theoretical yield of 100%. The starting materials are more accessible and allow the combination of a large pool of chemical units and substituents to form more complex molecular structures. Recently, aldolases have been shown to catalyze addition to activated ketones, enabling the stereoselective synthesis of tertiary alcohols.^[^ ^11^, ^12^ ^]^ However, acceptance of non‐activated ketones is limited and is currently being investigated mechanistically.^[^ ^13^ ^]^

YerE‐like enzymes are a class of thiamine diphosphate (ThDP)‐dependent enzymes that catalyze the asymmetric synthesis of tertiary alcohols using α‐keto acids and ketones as readily accessible substrates. *Yp*YerE and ErwE have been shown to catalyze aldehyde–ketone cross‐coupling reactions in vitro. Despite their biocatalytic potential, these enzymes show low donor substrate promiscuity, and their optimization has not been fully explored yet.^[^ ^9^, ^14^, ^15^ ^]^

JanthE is a recently characterized YerE‐like enzyme from *Janthinobacterium sp. HH01*, with an uncommon CDG ThDP‐binding motif and a broad spectrum of donor and acceptor substrates. JanthE accepts short to long α‐keto acids as donors, including pyruvate (**1**), 2‐oxobutanoate (**2**), 2‐oxovalerate (**3**), the sterically hindered 4‐methyl‐2‐oxovalerate (**4**) and the functionalized 4‐hydroxy‐2‐oxovalerate (**5**; with benzaldehyde). JanthE uses various ketones as acceptor substrates: both acyclic [phenoxy‐2‐propanone (**6**)], cyclic [β‐tetralone (**7**)], and linear [the 1,2‐diketone 3,4‐hexanedione (**8**)].^[^ ^16^ ^]^ Thus, JanthE can be used to obtain several tertiary alcohols with different branching lengths and sterically demanding groups. Although the physiological substrates have not yet been identified, the fact that the enzyme can accept a variety of non‐native substrates suggests even greater substrate promiscuity and versatility, making JanthE an interesting candidate for biocatalytic applications to produce chiral tertiary alcohols. Nonetheless, product formation is generally low, and optimization or further development of the enzyme is required for synthetic applications. Here, we investigate in detail the aldehyde–ketone cross‐coupling reactions catalyzed by JanthE. Focusing both on reaction parameters and residues of the active site involved in the catalysis, we develop the enzyme for better applicability.

## 2 Results and Discussion

### 2.1 Model Reaction and Wild Type Activity

**Figure 2:**
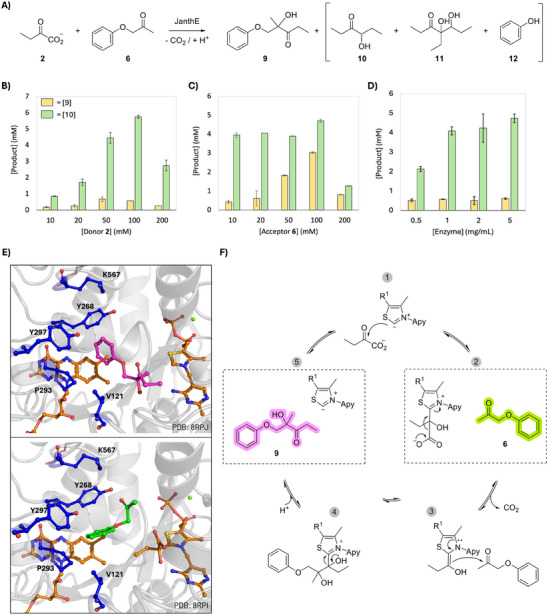
A) Reaction of 2 and 6 catalyzed by JanthE (side products in brackets). B–D) Concentration of the desired product 9 and side product 10 at alternating concentrations of donor 2 (B), acceptor 6 (C), or JanthE (D). Data are representative of duplicate experiments. E) Docking of acceptor substrate 6 (green) and product 9 (pink) in the active site. The critical amino acids selected for mutagenesis are shown in blue. The cofactors ThDP and FAD are shown as orange sticks and Mg^2+^ as a green sphere. F) Scheme of reaction steps catalyzed by JanthE. Steps 2 and 5 represent the snapshots tested by docking experiments. “Apy” stands for the aminopyrimidine ring and R^1^ is the diphosphate moiety of ThDP.

As a model reaction, we focused on the synthesis of the chiral 2‐hydroxy‐2‐methyl‐1‐phenoxypentan‐3‐one (**9**) (Figure 2A). This product is a sterically hindered aromatic compound that is not easily accessible by other YerE‐like enzymes or biocatalytic methods.^[^ ^14^ ^]^ JanthE catalyzes the addition of 2‐oxobutanoate (**2**) to phenoxy‐2‐propanone (**6**). Under the conditions tested (KPi 50 mM, DMSO 5%, pH 8, 50 mM donor, 20 mM acceptor, 1 mg·mL^−1^ purified enzyme, incubation at 28 °C for 16–20 h), 0.6 mM of product **9** (93% *ee*) was obtained. The low yields are partly due to the formation of additional unwanted products (Figure 2A). As a side reaction, the enzyme uses **2** in a homocoupling reaction to yield propioin (**10**). In addition, a second side product is formed both under the conditions of the reaction and when incubating the enzyme with **2** only. Based on GC–MS data, we suspect that this unknown product (**11**) results from a trimerization reaction of **2** (Figures S1 and S2). A similar behavior is observed when **3** is used as a substrate (Figure 5A and S3). However, assays performed with **3** and **10**, as well as **2** and butyroin (**14**) as substrates did not confirm our hypothesis. Compound **11** is neither commercially available nor easily accessible synthetically and therefore cannot be used as a control. Furthermore, the low product concentration makes NMR analysis to confirm the structure difficult. As an excess of donor substrate compared to the acceptor is typically used in our reaction setup, the characterization of side product **11** was neglected. Another side product found in the reaction is phenol (**12**) (Figures S1 and S2). Indeed, **12** is also found in the blank reaction (without enzyme) and does not significantly increase with time. Attempts to optimize the desired reaction (i.e., formation of **9**) focused on substrate and enzyme concentration.

Increasing the donor substrate concentration while maintaining [**6**] at 20 mm resulted in a decrease of product to propioin ratio, suggesting that the side rection is favored (Figure 2B). Vice versa, increasing the acceptor substrate concentration while keeping [**2**] at 50 mm increases the product **9** while increasing product to propioin ratio (Figure 2C). This shows that a higher acceptor to donor substrate concentration may be beneficial for the desired product formation. In turn, an increased enzyme concentration (from 0.5 to 5 mg·mL^−1^) did not improve the desired reaction (Figure 2D).

### 2.2 Engineering of JanthE Reveals Hotspot Residues

The lack of success in optimizing the reaction prompted us to opt for enzyme engineering. Docking studies were used to identify amino acids putatively involved in substrate or product binding (SI).^[^ ^16^ ^]^ We docked substrate **6** to the crystal structure of JanthE containing the pre‐decarboxylation‐bound intermediate KBThDP (pdb: 8rph) to mimic the conditions under which the acceptor substrate enters the active site before decarboxylation of the donor substrate. We also docked product **9** to the structure of JanthE in the resting state (pdb: 8rpj) to mimic the release of the product from the active site at the end of the reaction cycle (Figure 2E,F). Our docking experiments revealed five positions of interest that were further investigated by a combination of site‐saturation and site‐directed mutagenesis: V121, Y268, Y297, P293, and K567 (Figure 2E and Figures S4 and S5).

Y268 and P293 were previously suggested to play an important role in stabilizing the FAD in the active site for correct protein folding and limiting potential side reactions mediated by the redox cofactor.^[^ ^16^ ^]^ Mutating them to alanine, thus abolishing their structural roles, results in variants with reduced activity, hence confirming the proposed function (Figure S4). V121 was mutated to alanine, isoleucine, and methionine. While alanine at this position significantly decreases catalytic activity, V121M leads to a twofold increase in activity compared to the wild type (Figures S4 and S5). Isoleucine is an accepted residue at the same position as it negligibly increased product formation. Engineering the Y297 amino acid did not substantially affect the activity of the enzyme. Surprisingly, a proline at this position is accepted and does not interfere with the dynamics of the enzyme. The JanthE variants Y297P, Y297F, and Y297G exhibit a similar (Y297F and Y297G) or only slightly increased (Y297P) catalytic activity compared to the wild type under tested conditions (Figure S5). These results evidence that this amino acid is not essential for the catalytic activity of the enzyme, but it could be considered for supportive modifications aimed at increasing reaction yields.

Remarkable results were obtained with variants at position K567. The variants K567S and K567A increase product formation fivefold, with K567S resulting in 3 mM of product **9**. The increase in activity (*V_max _* = 0.030 ± 0.005 U·mg^−1^) is selective for tertiary alcohol formation; the product to propioin ratio increases from

**Figure 3:**
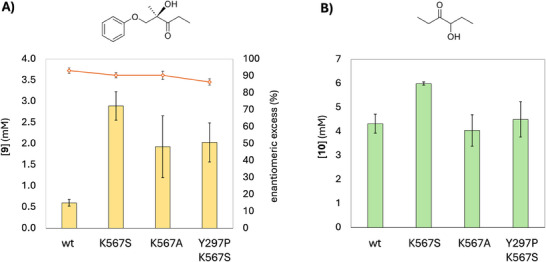
Concentration of product **9** A) and propioin **10** B) formed by wild type JanthE compared to single variants and selected double variant. The enantiomeric excess is shown as orange dots connected by a line. The data shown are the average of duplicate experiments.

∼0.2 of the wild type to ∼0.5 (Figure 3A). In addition, JanthE K567S and K567A still have good enantioselectivity (90% *ee* of **9**, (*R*)‐enantiomer based on CD spectra, see Figures S6 and S7).

The double variants at positions Y297 and K567 were also tested to account for possible positive combinatorial effects but were not substantially better with substrate **2** and **6** compared to variants at position K567 (Figure 3 and Figure S5). The double variant Y297P_K567S decreases product formation and enantioselectivity, compared to K567S, suggesting that the variation of Y297 is slightly detrimental under the tested conditions. Due to the higher enantioselectivity and catalytic activity the variant K567S was selected for further studies.

### 2.3 Kinetic Studies of JanthE Wild Type and K567S

**Figure 4:**
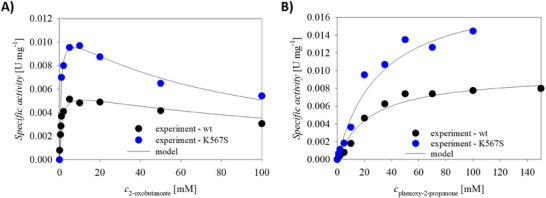
Dependence of the specific activity of JanthE wild type (wt) and variant JanthE_K567S (*c*_Mg2+_ = 1 mm, *c*_ThDP _= 0.05 mm, *c*_JanthE _= 1 mg·mL^−1^, 100 mm sodium phosphate buffer pH 8, *c*_NaCl _= 200 mm) on the concentration of A) 2‐oxobutanoate (*c*_phenoxy‐2‐propanone _= 20 mm) and B) phenoxy‐2‐propanone (*c*_2‐oxobutanoate _= 50 mm) for the formation of product **9**. Experimental data are representatives of triplicate experiments.

To compare the influence of the concentrations of donor and acceptor substrates on the specific activity of the wild type and the variant K567S enzymes for the formation of product **9**, basic reaction kinetics measurements were performed. Kinetic parameters were estimated from the experimental data presented in Figure 4A,B and are presented in Table 1. The details on the procedure are presented in SI. Both wild type and variant K567S do not show substrate inhibition by phenoxy‐2‐propanone (**6**) (Figure 4B). They show similar affinities to **6**: *K_m_* (**6**) of the wild type is 24.6 ± 3.8 mM and of the K567S variant is 31.8 ± 6.0 mM, respectively (Table 1). Concerning the donor substrate 2‐oxobutanoate (**2**), it has inhibiting properties on both the JanthE wild type and variant K567S. While the variant has higher affinities for the substrate (*K_m_* (**2**)* *= 0.74 ± 0.31 mM versus 0.92 ± 0.26 mM of the wild type) it also shows higher inhibition with a *K_i_* (**2**) of 97.5 ± 32.6 mM compared to the wild type with 156.5 ± 63.6 mM. Still, these constants are quite high indicating low substrate inhibition. The kinetic parameters for the side‐reaction, i.e., formation of **10**, at changing donor substrate concentrations were also assessed (Figure S9). For the wild type enzyme, the maximum reaction rate of the side reaction is lower than that for the formation of **9**, whereas for the K567S variant, the maximum reaction rate of the side reaction is similar to the main reaction. Additionally, the apparent Michaelis constants for 2‐oxobutanoate in the side‐reaction indicate that this reaction is less favored, as the constants are 5 and 20‐fold higher for wild type and variant, respectively, compared to the *Km* obtained for the product **9**. The substrate inhibitions are in the same order of magnitude for the main and side‐reactions. These findings can help guide further process development. Namely, substrate inhibition can be successfully mitigated by applying fed‐batch reactor mode and by calculating the rate of the substrate feed needed to facilitate maximum reaction rate. It can also help in minimizing the unwanted product concentration coming from the chemical background by adjusting proper reaction conditions.^[^ ^17^ ^]^

**Table 1:** Estimated kinetic parameters of JanthE wild type (wt) and K567S variant.

Parameter	Unit	Wt	K567S
*V_m_*	U mg^−1^	0.013 ± 0.002	0.030 ± 0.005
*K_m_* ^2‐oxobutanoate (^ **^2^** ^)^	mM	0.92 ± 0.26	0.74 ± 0.31
*K_i_* ^2‐oxobutanoate (^ **^2^** ^)^	mM	156.5 ± 63.6	97.5 ± 32.6
*K_m_* ^phenoxy‐2‐propanone (^ **^6^** ^)^	mM	24.6 ± 3.8	31.8 ± 6.0

### 2.4 Scalability and Versatility of JanthE Variants

Larger scale assays were performed in batch mode to assess the scalability of the enzymatic reaction catalyzed by the new variants [purified JanthE_K567S (1 mg·mL^−1^), **2** (50 mM), **6** (20 mM), 20 h, 28 °C]. Scalability was demonstrated in 5 mL and 15 mL reaction volume with purified enzyme, resulting in 2.5 mg and 6 mg of isolated product, respectively. In addition, to show applicability, the crude lysate was used in batch mode in 50 mL volume using an equimolar 50 mM concentration of donor **2** and acceptor **6**, resulting in 15 mg of isolated product. The production and isolation procedures are reported in SI. Our results confirmed the scalability of the reaction and the isolation of the desired chiral product (Figure S10). Further optimization, especially of the isolation procedure should be performed to increase the isolated yields.

**Figure 5:**
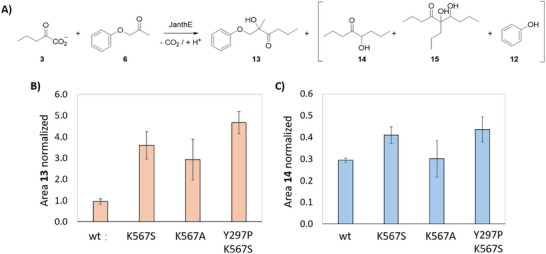
**A)** Reaction scheme with longer donor substrate **3**. **B)** Comparison of product **13** formed with JanthE wild type and selected variants. **C)** Comparison of the butyroin side product **14** formed with JanthE wild type and selected variants. The double variant Y297P_K567S showed the highest product formation. The data are representative of duplicate experiments.

To test whether the improvement in catalytic activity could be extended to other products, we investigated the variants JanthE K567S, K567A, and Y297P_K567S with the longer donor substrate **3** (Figure 5A). An almost fourfold increase in product formation compared to the wild type was confirmed to produce 2‐hydroxy‐2‐methyl‐1‐phenoxyhexan‐3‐one (**13**) using the K567S variant. Remarkably, the double variant Y297P_K567S gave a higher concentration of product **13** with a fivefold increase compared to the wild type (Figure 5A), demonstrating its value for biocatalytic applications. This variant has better performances with longer donor substrate **3** than **2,** therefore, it should be preferred if longer keto‐branching is the goal. Alcohol **13** is an even bulkier and more sterically hindered product than **9**, emphasizing the catalytic potential of the newly identified JanthE variants and selected hotspot residues.

### 2.5 Proposed Role of K567 and Y297 in JanthE

**Figure 6:**
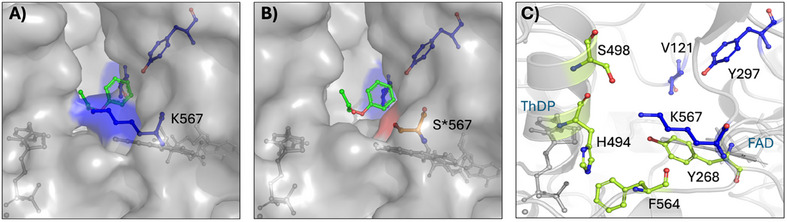
**A)** Surface view of K567 in the JanthE wild type from the perspective behind the active center. **B)** Surface view of S567 introduced by mutagenesis. Comparison of **A)** and **B)** shows that S567 opens a cleft into the active site. **C)** Selected amino acids in the vicinity of K567 that may play a similar role to K567S and are therefore potential targets for further mutagenesis studies.

The edge of the K567 and Y297 side chains are > 10 Å distant from the center of reactivity (Cα–C2–ThDP), thus being too far for a direct involvement in the reaction intermediates stabilization. More plausible is their role in substrates binding or product release. In some studies, the area characterized by K567 is referred to as the R‐pocket: the site of acceptor binding that determines (*R*)‐stereoselectivity in ThDP‐dependent enzymes.^[^ ^18^ ^]^ The variants K567S and K567A both show a slightly lower enantiomeric excess compared to the wild type. Similarly, introduction of proline at position Y297 in the double variant Y297P_K567S reduces enantioselectivity (Figure 3 and Figure S6). Nonetheless, as the exact site of acceptor binding in ThDP‐dependent enzymes is still elusive and may vary between different enzymes,^[^ ^18^ ^]^ we are careful in assigning such a role to the K567 or Y297 residues in JanthE without further crystallographic experiments. Surface visualization of the enzyme reveals that K567 outlines the active site cleft together with histidine 494 (Figure 6A,C). Mutation of lysine to smaller residues such as serine and alanine open a channel into the active site (Figure 6B). Therefore, the increased activity of K567S and K567A variants might be due to a beneficial effect of the introduced space. In support of this hypothesis, tests with a glycine at position 567 gave similar results to alanine and serine (data not shown). The nucleophilicity of the serine side chain could be important for the electrostatic balance in the active site compared to alanine, explaining the better conversions observed in our experiments. Supposedly, the channel formed by the helix hosting H494 and the loop hosting S467 may facilitate the binding of substrate **2** or **6**, release of product **9**, or allow favorable solvent interactions. Mutations targeting the residues F564, S498, and H494, located in closed proximity to K567 may provide further information about their specific function.

The better performance of the double variant Y297P_K567S suggests that the residue Y297 may be involved in donor substrate binding. The introduction of a smaller, although rigid, amino acid such as proline, at this position could favor a reduced steric effect of the donor **3** binding or the release of the more sterically hindered product **13** compared to **9**.

Mutations at position K567 and Y297P in other ThDP‐dependent enzymes have not yet been described, so it is difficult to speculate about the possible physiological roles in the wild type JanthE enzyme. Here, we show high relevance of these positions in the enzymatic catalysis for tertiary alcohols formation.

## 3 Conclusion

In this study, we have developed a novel enzymatic transformation for the enantioselective synthesis of sterically demanding tertiary alcohols. JanthE is versatile and evolvable for the desired reactions. A single point mutation (K567S) selectively increases the catalytic activity (*V_max_ K567S *= 0.030 ± 0.005 U·mg^−1^ versus *V_max_ wild type *= 0.013 ± 0.002 U·mg^−1^) for bulky tertiary alcohols resulting in a fivefold higher product formation and good (*R*)‐enantioselectivity (90% *ee*). The product, 2‐hydroxy‐2‐methyl‐1‐phenoxypentan‐3‐one, can be easily isolated from the reaction mixture. Additionally, the reaction is scalable for preparative synthesis. Preliminary studies performed with the double variant Y297P_K567S showed remarkable activity (fivefold product increase) with the long donor substrate **3**, further emphasizing the versatility of JanthE variants for various reactions.

Future studies could investigate scaling up the process and further enzyme engineering to improve productivity. A hot‐spot pocket in the active site of JanthE characterized by Y297, V121, and K567 was identified as a target to selectively increase the reaction yield in the formation of tertiary alcohols while keeping the benzoin‐type side activity unchanged. Mutations targeting other amino acids in the vicinity of K567 and double and triple variants in the identified pocket, should lead to optimization of the catalytic activity and enantioselectivity of the enzyme,^[^ ^18^, ^19^ ^]^ as has been shown in many studies on ThDP‐dependent enzymes.^[^ ^20^, ^21^ ^]^ As JanthE is known to have a broad substrate promiscuity, this research serves as a starting point for the biocatalytic application of this novel ThDP‐dependent enzyme for the synthesis of chiral tertiary alcohols.

## Supporting Information

The authors have cited additional references in the Supporting Information.^[^ ^22^, ^23^, ^24^, ^25^ ^]^

## Conflict of Interests

The authors declare no conflict of interest.

## Supporting information



Supporting Information

## Data Availability

The data that support the findings of this study are available in the supplementary material of this article.
