# Supplementary material for: Engineering of the Thiamine Diphosphate‐Dependent JanthE for the Synthesis of Tertiary Alcohols
Source: Chemistry. 2025 Jun 8;31(35):e202500890. doi: 10.1002/chem.202500890 (PMC12188165; doi:10.1002/chem.202500890)
Supplement: Supplementary file 1 — Supporting Information [file CHEM-31-e202500890-s001.docx]

Supporting Information

# **Engineering of the Thiamine Diphosphate-Dependent JanthE for the Synthesis of Tertiary Alcohols**

Lucrezia Lanza^a^, Daniela Bjarnesen^a^, Mehmet Mervan Çakar^b^, Andrea Rizzo^c^, Dr. Akash Pandya^c^,
Dr. Carmen Aranda^c^, Prof. Dr. Zvjezdana Findrik Blažević^b^, Prof. Dr. Michael Müller^a^

[a] L. Lanza, D. Bjarnesen, Prof Dr. M. Müller
Institute of Pharmaceutical Sciences
Albert-Ludwigs-Universität Freiburg
Albertstrasse 25, 79104 Freiburg, Germany
E-mail: michael.mueller@pharmazie.uni-freiburg.de

[b] Dr. M. M. Çakar, Prof. Dr. Z. Findrik Blažević
University of Zagreb, Faculty of Chemical Engineering and Technology
Trg Marka Marulića 19, Zagreb, Croatia

[c] Dr. A. Rizzo, Dr. A. Pandya, Dr. C. Aranda
Johnson Matthey
Milton Road CB4 0FP, 28 Cambridge Science Park, Cambridge, United Kingdom

**Enzyme JanthE**

The JanthE wild type enzyme from *Janthinobacterium sp. HH01* was previously characterized biochemically and structurally.^[16]^ The enzyme is a homodimer that belongs to the YerE-like enzymes subclass of ThDP-dependent decarboxylases. It tighly binds both ThDP and FAD in the active site. Only ThDP is catalytically active. FAD is an evolutionarily relict conserved from the pyruvate oxidase anchestor that is needed for correct folding. Expression and purification methods followed the same approaches as specified in reference [16], if not otherwise stated here.

**Chemicals and Reagents**

Chemical reagents and solvents were purchased from Sigma–Aldrich (St. Louis, USA), AppliChem (Darmstadt, Germany), Roth (Karlsruhe, Germany), Alfa Aesar (Karlsruhe, Germany), or BLD Pharmatech Ltd. (Shanghai, China) at the best possible purity. Buffer A (K_2_HPO_4_ 50 mM, KH_2_PO_4_ 50 mM, NaCl 200 mM, MgCl_2_ x 6 H_2_O 1 mM, ThDP 50 μM) was used for enzymatic assays.

**Strains and cultivation conditions**

*Escherichia coli DH5α* was used for cloning and plasmid propagation. *E. coli BL21 (D3)* was used for enzyme expression unless otherwise stated. The strains were cultivated and selected in LB media supplemented with ampicillin (50 μg·mL^–1^).

**JanthE wild type nucleotide sequence.** Retrieved from NCBI with locus_tag JAB_RS30350. Nucleotides added in the cloning process are shown in red.

ATGATGCAAGACAATAAAGTCAAGGTCGCCGAACTGGTGGCCGAAGCCCTGGAAAACCTGGGCATCCAGCACGCCTTCGGCATCATCGGCGCCGGCAATGTGCACCTGTTCGAAGCCATCGCCCGCCGCGGCTACACCGAGATCGTCTGCGTCCACCACGAGCAGGCAGCGTGCATGGCGGTGCAGACCTACTACCGCACCAACGGCCGCATCGCCGCCGCGCTGCTGACCACCGGCGCCGGTTCGACCAATGGCGTCACCGGCGTGGTGTCGGCGTGGGCCGACTCGATTCCGTGCATCGTCATCGCCGGCAACGAGAACTCCAAGTTCACCTTCCCGGAAAACCCGCTGCGCATGTGGGGCGTGCAGGGCTACGATTCCTGCCAGATGGTGGAACGCGTCAGCAAGTACCAGATGCGCGTGACGAAGATGGAACGCGCCGTCTACGAACTGGAAAAAGGCGTGCACCTGGCGCTGGAAGGCCGTCCGGGTCCGACCTGGATCGAGATCCCGATGGACATACAGTCCGGCCGCATCGATCCGGCCACGCTGGAACACTACGTCGCCCCGCCCGCACCCGACTACCTGACGCCGGCCGTGGCCGCGCAGGTGGACAGCGTGCTGGCCGCCCTGGCAAAGGCCGAGCGTCCGGTGCTCTGGCTGGGCAACGGCATCCGCCTGGCCGGCGGCGAGCGTCTGCTCAAGCCGCTGCTGGAAAAACTCGGCTCGCCGGCGCTGGTGTCGTGGGCCGGCATCGACATGCTCGACTCCAGCCACCCGCTGGTGTTCGGCCGCGCCGGCGTCTACGGCCAGCGCGCCGCCAACTTCATTTTGCAGAACAGCGACTACGTGCTGGCCATCGGCACCCGCCTGGCGATCCCGCAGATCGGCTACGACCTCAATGAGCTGGCGCGCCTGGCGCGCATCGACGTGGTCGACATCGACGGCGACGAGGCGATCAAGCACGCCAAGCGCACGCAGGAAAACATCGTCTGCGACGCCCGCGTCTTCATCGAGGCGCTGCTGGCCCGCCTCAACGCCGCCGACGCCCCGGCCATCGCCTCCAAGGCCGACTGGGTCGCCAAGTGCCGCGCCTACGAGGAGCAGTTCCCGTGGGTGGGCGCCGAGCACGCGGACCCGGAAGGTTTCATCAACTCCTACCGCTTCATGGAGCGCCTGAACGGCTTCTTCAAGGACGACCAGGTGGTCGTCACCGACATGGGCACGGCGCTGCTGAGCGGCCACCAGGTGCTGCGCTTCAAGGAAGGCCAGCGCTTCATGACCTCCACCGGCCTGGGCGAGATGGGCTACGGCCTGCCGGCCGCGCTGGGCGTATCGTTCGCCAACGACCGCGGCGAGGTGATGTGCCTGAACTGCGACGGCGGCATGATGATGAATCTGCAGGAGCTGCAGACCATGGTGCACCACAACCTGCCGATCAAGCTGTTCATCTTCAACAACGACGGCTACCTGATGATCAAGCACACGCAGAAGTCGCTGTTCAAGTCGGACTACGTCGGCACCGACCGCAAGTCCGGCGTGTCCTGCCCCGACTTCTCCAGGCTGGCCGCCGCCTTCGACATCCCCGCCTACCAGATCCGCGGCTGGGACGAGTGCGACGCCACCTTGGCCAAGGTGCAGGCGCACACCGGCCCGGTGATCTGCGAGGTGTTCATGCATCCGCAGCAGCTGTTCTCGCCGAAGCTGGGCGTGGTCTCGCGCGCCGACGGCACGCTGGTGTCGCCGCCGCTGGAAGACCTGTCGCCGCTGATCCCGCGCGATGTGCTGGAGCAGGCCATGATAGGCGGCATGCACGAGAAGTCGAAAACGCTCCTCGAGCACCACCACCACCACCAC

**JanthE wild type amino acid sequence.** Protein id WP_008453236.1. All the additional residues introduced with cloning are shown in red. Position investigated in this study through engineering are reported in bold and blue.

MMQDNKVKVAELVAEALENLGIQHAFGIIGAGNVHLFEAIARRGYTEIVCVHHEQAACMAVQTYYRTNGRIAAALLTTGAGSTNGVTGVVSAWADSIPCIVIAGNENSKFTFPENPLRMWG**V**QGYDSCQMVERVSKYQMRVTKMERAVYELEKGVHLALEGRPGPTWIEIPMDIQSGRIDPATLEHYVAPPAPDYLTPAVAAQVDSVLAALAKAERPVLWLGNGIRLAGGERLLKPLLEKLGSPALVSWAGIDMLDSSHPLVFGRAGV**Y**GQRAANFILQNSDYVLAIGTRLAI**P**QIG**Y**DLNELARLARIDVVDIDGDEAIKHAKRTQENIVCDARVFIEALLARLNAADAPAIASKADWVAKCRAYEEQFPWVGAEHADPEGFINSYRFMERLNGFFKDDQVVVTDMGTALLSGHQVLRFKEGQRFMTSTGLGEMGYGLPAALGVSFANDRGEVMCLNCDGGMMMNLQELQTMVHHNLPIKLFIFNNDGYLMIKHTQKSLFKSDYVGTDRKSGVSCPDFSRLAAAFDIPAYQIRGWDECDATLAKVQAHTGPVICEVFMHPQQLFSP**K**LGVVSRADGTLVSPPLEDLSPLIPRDVLEQAMIGGMHEKSKTLLEHHHHHH

**Primer used in the study**

Primers were purchased from Eurofins Genomics (Ebersberg, Germany). The delivered stocks were used at a working concentration of 10 µM. Bold nucleotides correspond to the engineered positions.

| **Primer name** | **Sequence 5´–3´** |
| --- | --- |
| Site-directed mutagenesis | |
| J_Y297F_rv | GAGGTC**GAA**GCCGATCTGCGGGATCGCC |
| J_Y297F_fw | GATCGGC**TTC**GACCTCAATGAGCTGGCGCG |
| J_Y297E_rv | GAGGTC**TTC**GCCGATCTGCGGGATCGCC |
| J_Y297E_fw | GATCGGC**GAA**GACCTCAATGAGCTGGCGCG |
| J_Y268H_rv | CTGGCC**GTG**GACGCCGGCGCGGCCGAAC |
| J_Y268H_fw | GCGTC**CAC**GGCCAGCGCGCCGCCAACTTCATTTTG |
| J_Y268A_rv | CTGGCC**TGC**GACGCCGGCGCGGCCGAAC |
| J_Y268A_fw | GCGTC**GCA**GGCCAGCGCGCCGCCAACTTCATTTTG |
| J_V121A_rv | GCCCTG**TGC**GCCCCACATGCGCAGCG |
| J_V121A_fw | GTGGGGC**GCA**CAGGGCTACGATTCCTGCCAG |
| J_P293A_rv | CCGATCTG**TGC**GATCGCCAGGCGGGTG |
| J_P293A_fw | GGCGATC**GCA**CAGATCGGCTACGACCTCAATG |
| J_K567S_rv | GCCCAG**CGA**CGGCGAGAACAGCTGCTGC |
| J_K567S_fw | CTCGCC**GTC**GCTGGGCGTGGTCTCGC |
| J_K567A_rv | GCCCAG**TGC**CGGCGAGAACAGCTGCTGCGGATG |
| J_K567A_fw | CTCGCCG**GCA**CTGGGCGTGGTCTCGCGCG |
| J_V121M_fw | GTGGGGC**ATG**CAGGGCTACGATTCCTGCCAG |
| J_V121M_rv | GCCCTG**CAT**GCCCCACATGCGCAGCG |
| J_V121I_fw | GTGGGGC**ATC**CAGGGCTACGATTCCTGCCAG |
| J_V121I_rv | GCCCTG**GAT**GCCCCACATGCGCAGCG |
| Site-saturation mutagenesis | |
| J_121V_fw | CCCTGKNNGCCCCACATGCGCAGCGGGTTTTC |
| J_121V_rv | GTGGGGCNNMCAGGGCTACGATTCCTGCCAG |
| J_268Y_fw | CGCTGGCCKNNGACGCCGGCGCGGCCGAACACC |
| J_268Y_rv | GCGTCNNMGGCCAGCGCGCCGCCAACTTC |
| J_293P_fw | GATCTGKNNGATCGCCAGGCGGGTGCC |
| J_293P_rv | GCGATCNNMCAGATCGGCTACGACCTC |
| J_297Y_fw | GAGGTCKNNGCCGATCTGCGGGATCGCCAGG |
| J_297Y_rv | CAGATCGGCNNMGACCTCAATGAGCTGGC |
| J_567K_fw | CCCAGKNNCGGCGAGAACAGCTGCTGCGGATG |
| J_567K_rv | CTCGCCGNNMCTGGGCGTGGTCTCGCG |

**Mutants generation by PCR**: The plasmid pet22b bearing the JanthE wild type gene with a 6-His tag at the C-terminal end was used as a template. PCR amplification was performed using Phusion Flash High-Fidelity 2x Master Mix (Thermo Fisher Scientific, Waltham, USA) with 3% dimethyl sulfoxide (DMSO) and a template DNA diluted from its original mini prepped concentration 1:40. PCR was run in a final volume of 50 μL on a Primus 25 advanced thermocycler (Peqlab, currently, VWR International, Germany) for 30 cycles using a melting temperature of 72 °C and extension time of 2.40 minutes. A final elongation of 72 °C for 10 minutes was used to ensure complete plasmid propagation. Samples were treated with 1 µL of DpnI restriction enzyme (NEB, Ipswich, USA) for parental plasmid degradation and incubated at 37 °C for 1 h. Subsequently, samples were loaded onto a 1% [w/v] agarose gel with a 2-log 1kb DNA ladder (NEB, Ipswich, USA). Electrophoresis was performed in TAE buffer at 70 mV for 45 min. The gel was stained in ethidium bromide solution (6.6 mg·L^–1^) and DNA samples were detected at 360 nm (UV-Transluminator Bio-Vision, Vilber Lourmat, Marne-la-Vallée, France). In the case of site-directed mutagenesis of position Y268, the PCR gel showed unspecific amplification in addition to the wanted fragment size at 7.2 kb, therefore, the fragment with the correct size was cut from the gel and purified with the Nucleospin® Gel and PCR cleanup kit (Macherey-Nagel, Germany). Purification of the other plasmids was carried out directly using the PCR solution. In the case of site saturation mutagenesis of Y268, the PCR was unsuccessful, therefore, the mutation was not further explored.

**Cloning and sequencing**: Transformation of *E. coli* DH5α chemical competent cells was performed by heat shock following standard protocols and plated on LB media supplemented with 50 μg·mL^–1^ ampicillin. Plasmid isolation was conducted from 10 mL overnight culture using the GeneJet Plasmid MiniPrep kit (Thermo Fisher Scientific, Waltham, USA). Nucleotide concentration was measured with a NanoDrop 2000 Spectrophotometer (Thermo Fisher Scientific, Waltham, USA). The correctness of the mutations for site-directed mutagenesis and the mutation inserted with site saturation mutagenesis that gave positive results were confirmed by Sanger sequencing performed by Mycrosynth AG (Balgach, Switzerland).

**Site saturation mutagenesis workflow**: Y297 and V121 PCR products digested with DpnI were transformed in *E. coli DH5α*. After overnight growth, the colonies on the plate were collected with LB media, and the collective plasmid was isolated with the GeneJet Plasmid MiniPrep kit (Thermo Fisher Scientific, Waltham, USA). The purified plasmid sample was used for transformation in *E. coli* BL21 (D3). For the mutants K567 and P293 a different workflow was followed. PCR products digested with DpnI were transformed in *T7 Express Competent E. coli* cells (NEB, Ipswich, USA). For all the mutations, single colonies of the respective expression strain were transferred in a 96-well plate with 500 μL of LB and grown overnight at 37 °C, 200 rpm in an Ecotron Incubator Shaker (Infors HT, Switzerland). Each plate had a positive control eg., JanthE wt to test the activity of variants versus the wild type. Negative control e.g., *E. coli* bearing an empty pet22b vector was used to account for the activity of other enzymes in the cell-free extract of *E. coli*. Finally, wells with solely LB were used to monitor for evaporation and contamination among the wells. 10 μL of overnight pre-culture was added to a new 96 well plate with 1 mL LB medium supplemented with 50 μg·mL^–1^ ampicillin and grown for 3 h at 37 °C, 200 rpm, before induction with 0.2 mM isopropyl-β-D-thiogalactopyranoside (IPTG). Expression was conducted at 25 °C, 200 rpm for 16 h. The remaining of the pre-cultures was used for glycerol stocks (25% v/v glycerol) that were stored at –20 °C. Harvesting was performed by centrifugation for 30 min at 4 °C and 8000 rpm, (Centrifuge 5804 R and A-2-DWP rotor, Eppendorf, Hamburg). LB media was discarded, and the pellet was resuspended in 250 μL of assay solution (Buffer A at pH 8, 50 mM 2-oxo butanoate, and 20 mM phenoxy-2-propanone). The plate was vortexed for a few minutes and incubated at 28 °C, 200 rpm for 16 h. Two rounds of extraction were performed by adding 600 μL of ethyl acetate. After each round of extraction, the plate was vortexed for 10 seconds and centrifuged at 3500 rpm for 10 min. 500 μL of the organic phase was transferred to a separate 96 well plate.
100 µL of the accumulated 1 mL extracted volume was used for GC-MS analysis. As everything was conducted reducing at minimum the variation between samples, a hit was chosen when the peak of the product was higher than the wild type. Selected hits were transferred from the glycerol stock at
–20 °C into 5 mL of LB media supplemented with 50 μg·mL^–1^ ampicillin for overnight pre-culture. The next day, 500 µL of pre-culture was transferred in 50 mL of LB supplemented with 50 μg·mL^–1^ ampicillin, 24 μM thiamine hydrochloride (ThCl), and grown at 37 °C, 170 rpm in a 250 mL Erlenmeyer flask. After 3 h of growth, the culture was induced with 0.2 mM of IPTG and expression was conducted at 25 °C for 16–18 h. The culture was transferred in a pre-weighted 50 mL falcon tube and harvesting was performed by centrifugation for 30 min at 4 °C and 5000 rpm (Megafuge ST plus series, Thermo Scientific, USA). The supernatant was discarded, and the pellets were stored at –20 °C until further use. 15 mL of Buffer A per g of pellet was added to ensure comparable enzymatic dilution. Sonication was performed with a flat tip, three times, 30 s, 30 s break in between, duty cycle 50%, output control 5 (Branson Sonifier II W-250, Heinemann, Schwäbisch Gmünd, Germany). The cell debris was sedimented by centrifugation for 30 min at 4 °C and 5000 rpm (Megafuge ST plus series, Thermo Scientific, USA). The supernatant [(e.g., cell free extract (CFE)] was transferred to a new tube and stored at 4 °C until in vitro assay was performed.

**Purification of wild type enzyme and variants:** This step followed the same methodology as described elsewhere.^[16]^ The only differences are the use of *E. coli BL21* strains as expression system and incubation at 25 °C for expression. In addition, sonication was performed with a flat tip, 4 times, 30 s, 30 s break in between, duty cycle 50%, output control 5 (Branson Sonifier II W-250, Heinemann, Schwäbisch Gmünd, Germany).

**Small scale analytical assays**: In vitro assays were performed in Buffer A at pH 8 with a total volume of either 250 or 500 µL. The phenoxy-2-propanone (**6**) was dissolved in DMSO, while the donor substrate 2-oxobutanoate (**2**) or 2-oxovalerate (**3**) were dissolved in Buffer A, to reach a stock concentration of 400 mM and 500 mM, respectively. From the prepared stock of acceptor substrate, a volume to reach max 5% DMSO concentration in the assay was added to the assay sample. When using CFE an assay volume of 500 µL was used with 425 µL of CFE, 50 µL of **2,** and 25 µL of **6**. CFEs resulting from empty vector of *E. coli* BL21 (for V121 and Y297) or *T7 Express* (K567S and P293) were used as negative controls when performing the assays for the site saturation mutagenesis experiment. When purified protein was used, the protein stock was added to the assay to reach a final concentration of 1 mg·mL^–1^. When all the components were added together, assays were quickly vortexed and spinned down, and incubated at 28 °C and 900 rpm 16–48 h as specified for each experiment. A solution of ethyl acetate and 2.5 mM toluene was used to extract the non-polar products. Toluene was used as internal standard accounting for extraction differences. Two rounds of extraction were performed by adding 300 µL in case of 250 µL assay volume (assay A) and 500 µL in case of 500 µL assay volume (assay B). After each round of extraction, the tube was vortexed for 10 s and centrifuged at 3500 rpm for 3 min. 250 µL for Assay A and 450 µL for assay B of organic phase was transferred to a new Eppendorf tube 1.5 mL. In the second round of extraction 300 µL for assay A and 500 µL for assay B, were transferred, respectively. The extracted solution was used directly for analysis. Analytical assays performed using 200 mM of either donor or acceptor substrate concentration were performed with a buffer A modified to have 100 mM K_2_HPO_4_ and 100 mM KH_2_PO_4_ (Figure 2B and 2C). Analytical assays performed with **3** and **6** were performed in the same way as described above in a 250 µL assay volume. Gas Chromatography/Mass Spectrometry (GC/MS) was used as analytical method to compare the activity of variants, GC was used for kinetics experiments, and HPLC chiral was used to estimate enantiomeric excess.

**Larger scale analytical assay with K567S variant:** 5 mL and 15 mL assay were performed only with purified JanthE K567S. The preparation of stocks and volumes of substrates to add followed the same method as described for smaller analytical scale assays. The assay was conducted in a bottom round flask of 100 mL for the 5 mL assay and 250 mL for the 15 mL assay. The assay was incubated at 28 °C and 170 rpm in an Ecotron Incubator Shaker (Infors HT, Switzerland) for 24 h. Ethyl acetate was used for extraction. Double the volume of the assay volume was used for three times extraction. A precipitate of the enzyme was formed after the reaction was complete, therefore, particular care was taken to not transfer the precipitate in the extracted organic phase. A separatory funnel was used to facilitate phase separation, and the ethyl acetate organic phase was transferred in a new round bottom flask using a syringe and needle. Evaporation was conducted with a Laborota 4000 rotor evaporator (Heidolph 2, Germany) using a bath at 40 °C and a pressure of 230 bar to remove the ethyl acetate. When the solution was evaporated to approximately 1 mL, it was transferred into an Eppendorf tube of 1.5 mL, and evaporated with Speed Vacuum concentrator (Eppendorf 5301, Hamburg, Germany). When ethyl acetate was completely evaporated, 1 mL of acetonitrile was added in preparation for HPLC separation.

**Enzyme kinetics:** The influence of concentrations of donor (**2**) and acceptor (**6**) substrates on enzyme activity was evaluated independently by using the initial reaction rate method (e.g., substrate conversion below 10%). Experiments were conducted keeping constant each reaction conditions while changing either the donor or the acceptor substrate concentrations. While the concentration of 2‑oxobutanoate was varied, the concentration of phenoxy-2-propanone was kept constant at 20 mM. While the concentration of phenoxy-2-propanone was varied, the concentration of 2-oxobutanoate was kept constant at 50 mM. Assay conditions used were as follow; 1.5 mL Eppendorf tubes, Buffer A, pH 8, reaction volume of 250 µL using DMSO 5%, and purified enzyme (either K567S variant or wild type) at a concentration of 1 mg∙mL^−1^. The assays were incubated at 28°C and 900 rpm. Ethyl acetate with 5 mM of toluene was used for stopping the reaction at selected times, and extraction followed the same procedure as described before for small analytical assays. GC was used as analytical method. 5–6 time points were collected for each experiment. Kinetics parameters reported in Figure 4 and Table 1 were calculated based on the product 2-hydroxy-2-methyl-1-phenoxypentan-3-one (**9**) formation. The enzyme activity was calculated based on the change of product **9** concentration in time according to the equation S1. A calibration curve of product was constructed based on the purified product obtained in larger scale production experiment using K567S as reported above.

$S.A.=\frac{\text{d}c_{\text{2-hydroxy-2-methyl-1-phenoxypentan-3-one }(\mathbf{9})}}{\text{d}t}\cdot\frac{V_{\text{r}}}{V_{\text{JanthE}}}\cdot\frac{1}{\gamma_{\text{JanthE}}}\text{ }\left[\frac{\text{U}}{\text{mg}}\right]$ (Eq S1)

One Unit of JanthE activity was defined as the amount of enzyme needed to catalyze the formation of 1 µmol of product 2-hydroxy-2-methyl-1-phenoxypentan-3-one (**9**) per minute at 28 °C in 100 mM potassium phosphate buffer, pH 8.0.

The experimental data on the dependence of the specific enzyme activity on the concentration of donor and acceptor substrate was used to estimate the kinetic constants. For this purpose, program package, SCIENTIST and non-linear regression analysis were used. Simplex and Least squares methods implemented in SCIENTIST were used for the estimation of the kinetic parameters. The same software was used for the simulation of data presented in Fig 4. Single substrate kinetic equation was used to estimate the initial values of the next step, i.e., estimation of parameters by using the double substrate kinetics and obtained unique maximum reaction rate (*V_m_*). Equation S2 was used for the estimation of *K_M_*, *K_i_* , and *V_m_* values meaning that both series of data were used simultaneously in the second round, i.e., *c*_2-oxobutanoate_, *c*_phenoxy-2-propanone_ and specific activity data.

$S.A.=\frac{V_{m}\cdot c_{\text{phenoxy-2-propanone}}\cdot c_{\text{2-oxobutanoate}}}{\left(K_{m}^{\text{phenoxy-2-propanone}}+c_{\text{phenoxy-2-propanone}}\right)\cdot\left(K_{m}^{\text{2-oxobutanoate}}+c_{\text{2-oxobutanoate}}+\frac{c_{\text{2-oxobutanoate}}^{2}}{K_{i}^{\text{2-oxobutanoate}}}\right)}$ (Eq S2)

Kinetics parameters reported in Figure S9 were calculated based on the formation of propioin (**10**) and only depending on donor substrate concentration. The same approach used for product **9** was used to calculate parameters for propioin (**10**).

**Gas Chromatography/Mass Spectrometry (GC/MS)**: The 8890 GC System equipped with 5977C Mass Selective Detector and with corresponding Injector and Autosampler from Agilent Technologies (Santa Clara, USA) was used for GC/MS analysis. The system was equipped with a HP-5MS UI column (length = 30 m, diameter = 0.25 mm, film = 0.25 µm) from Agilent Technologies (Santa Clara, USA). Helium with a constant flow of 1.0 mL∙min^−1^ was used as the carrier gas and 1 µL of sample was injected for each run. Inlet was operated with helium in split mode (41.7:1) with a heater temperature of 250 °C, 0.566 bar, and 41.6 mL∙min^−1^ split flow. The method featured a gradient with a slope of 20 °C∙min^−1^ from 3 to 14 min: T_0_ min = 60 °C, T_3_ min = 60 °C, T_14_ min = 280 °C, T_19_ min = 280 °C. The product **9** eluted at 10.32 min while product **13** at 8.7 min. A calibration curve of product **9** and propioin **10** was constructed for data analysis and comparison.

**Chiral phase gas chromatography (GC):** Chiral phase GC-FID was carried on a 2100 instrument (Shimadzu, Kyoto, Japan) equipped with an FID and a MEGA-DEX DMP Beta column (MEGA s.n.c., Legnano, Italy; length 25 m, inner diameter 0.25 mm, film thickness 0.15 μm). Helium was used as carrier gas with pressure control mode at 0.80 bar with a total flow of 76.2 mL∙min^–1^. The injection volume was 1 µL with a split ratio of 66.8:1 Ionization was performed by flame ionization at 250 °C with an H_2_ flow of 40.0 mL∙min^–1^ and airflow of 400.0 mL∙min^–1^ setting a sampling rate of 40 Hz. The temperature program was run with a gradient of 10 °C∙min^–1^ from 3 to 14 min: T_0_ min = 60 °C, T_3_ min = 60 °C, T_14_ min = 190 °C, T_17_ min = 190 °C. The product **9** eluted at 16.2 min. A calibration curve of product **9** and propioin **10** was constructed for data analysis and comparison.

**Chiral-phase High-Performance Liquid Chromatography (Chiral-HPLC)**: was performed on an Agilent 1100 chromatography system (Agilent Technologies, Santa Clara, USA), equipped with a photodiode array detector and Agilent 1260 series degasser. A Chiral OD-H column (250 · 4.6 mm), with guard column (Daicel, West Chester, USA) was used for the identification of enantiomeric excess of compound **9**. Each analysis was run at 25 °C, 0.9 mL∙min^–1^, n-hexane/2-propanol = 98:2 for 25 min.
2 µL of sample was used for injection. The assays for HPLC chiral analysis were conducted in 250 µL volume, extracted two times with 300 µL and evaporated to 100 µL in a Speed Vacuum concentrator (Eppendorf 5301, Hamburg, Germany). Full UV spectra was recorded, and specific wavelength used were: 210, 225, 230, 254, and 270 nm. The product *rac*-**9** chemically synthesized was used as a reference to identify (*S*)- and (*R*)-product peaks. **6** was also run as control because after the assay much of **6** was still left in the vial and the peak was visible in the chromatogram.

The UV – spectra of the product shows peaks at 225 and 270 nm (Figure S5). When analyzing the chromatogram of the wild type and the variants, with the 225 nm analysis, a double peak was visible for the less represented enantiomer, while it was not visible for the 270 nm analysis. For this reason, the 270 nm wavelength was used for enantiomeric excess calculation.

**High-Performance Liquid Chromatography (HPLC):** was performed to isolate the product *rac*-**9** from chemical synthesis or (*R*)-**9** from JanthE K567S larger scale production. An Agilent 1260-90 Infinity II System equipped with a quaternary pump in combination with an ISAspher 100-5-C18 Aq column (250×4.0 mm) (Isera Gmbh, Germany) was used. The method was run for 2 min with 50% acetonitrile (ACN) and water, followed by a gradient of 20 minutes reaching 100% ACN, 10 min run at 100% ACN, and 5 min gradient back to 50% acetonitrile. The semi-preparative scale product purification was run using 100 µL of injection volume. A total of 10 injections were needed to run the full samples (e.g., either extracted from 5 mL or 15 mL, or chemical synthesis). Each fraction containing **9** was evaporated of acetonitrile, combined, and extracted with three rounds of 600 µL ethyl acetate. Lyophilization was tried to reduce losses but was unsuccessful. A pre-weighted Eppendorf was used for evaporation of the ethyl acetate by Speed Vacuum concentrator. After complete evaporation the tube was weighted to determine the isolated yield of pure product formation and stored in the fridge until further use. Tests with GC/MS confirmed the purity of the sample.

**Circular dichroism (CD)**: CD spectroscopy was performed on a Jasco J-810 spectrometer (Jasco, Tokyo, Japan). Measurement was performed at 20 °C with acetonitrile as solvent and a cuvette with cell pathlength of 1 mm. Sample was extracted with ethyl acetate as described before and completely evaporated before addition of acetonitrile. Either 0.5 µL or 2.5 µL of isolated product (from the K567S production) was resuspended in 300 µL of acetonitrile for the measurement. Data represent the accumulation of three single measurements, bandwidth was 1 nm and scanning speed was
50 nm·min^–1^. Spectra of pure solvents were measured and subtracted from the values of the sample. Spectra were recorded from 210 to 400 nm. A negative cotton effect was recorded at 295 nm. When increasing product concentration, the larger peak showed a broader maximum negative peak spanning between 290–300 nm (Figure S7).

**Nuclear Magnetic Resonance spectroscopy**: NMR spectra were recorded in CDCl_3_ on a 400 MHz Bruker Avance III HD spectrometer. Chemical shifts are referenced to the residual solvent peak. The following multiplicity abbreviations are used: (s) singlet, (d) doublet, (t) triplet, (m) multiplet.

**Chemical synthesis and purification of 9:** Chemical synthesis of product **9** was performed following the protocol reported by Dr. Lehwald in her dissertation but using propionyl chloride as substrate instead of acetyl chloride.^[22]^ Briefly, 50.0 mL of SmI_2_ (0.1 mol in THF) was placed in a round bottom flask under nitrogen atmosphere. A mixture of 0.21 mL of propionyl chloride (2.4 mmol, 1.06 g·mL^–1^) and 0.33 mL phenoxy-2-propanone (2.4 mmol, 1.097 g·mL^–1^) in 6 mL of dried THF was rapidly added at room temperature. The solution quickly turned from dark blue to yellow. It was let run for other 10 minutes before 24 mL of 0.1 M HCl was added. Subsequently, 30 mL diethyl ether was added to separate the organic and aqueous phases. The aqueous phase was then extracted again with 3 x 50 mL of diethyl ether. The combined organic phase was dried with anhydrous MgSO_4_ followed by evaporation on a Laborota 4000 rotor evaporator (Heidolph 2, Germany) using a bath at 40 °C and a pressure of 230 bar. Isolera Prime Flash Purification System (Biotage, Uppsala, Sweden) was used for purification together with hand-packed Biotage SNAP Ultra 10 g columns. The column bed was a silica gel 60 (particle size: 40–63 μm; Merck, Darmstadt, Germany) pre-treated with an acidic solution for better separation of the wanted product. The purification was run with Cyclohexane/ Ethyl acetate 5:1. Fraction containing the desired product was not pure, therefore further purification with HPLC semi-preparative system was conducted as described above. The synthesis and purification methods described here resulted in 1 mg of pure product corresponding to an isolated yield of 0.2 % considering phenoxy-2-propanone the limiting reagent.

**Docking:** The available structures of JanthE pdb: 8rpj and pdb: 8rph were used as models for docking of the product (**9**) and acceptor (**6**) substrate, respectively. Molecular docking was carried out using Glide within the Schrodinger 2022.1 software suite. The Protein Preparation Wizard tool was applied to the JanthE crystal structure to add hydrogens and perform energy minimization with the OPLS4 forcefield. The substrate and product structures were prepared using the LigPrep tool. A search grid was setup around the enzyme active site to facilitate the molecular docking. A total of ten docked poses for the substrate and the product were generated and the lowest energy pose was selected for further analysis. An in-house custom Python script was used to analyze the contacts.

**Data and statistical analysis:** Analysis of GC-MS data was conducted using the MassHunter Qualitative Analysis software (Agilent Technologies). The area identified by the software corresponding to each peak of interest was used for further analysis. The raw area was normalized with the area corresponding to the toluene peak to account for extraction errors. The equation found through calibration curves (with R^2^ > 0.99) was used to calculate compound concentration. The data are average of experiments conducted in duplicate or triplicate. Standard deviation is reported as error bar. The same approach was used for GC-FID analysis. Analysis of HPLC-chiral data was performed using Agilent Chemstation software. The area under the curve was used for enantiomeric excess calculations. Data are representative of three different experiments. CD spectra were formatted for figure representation with spectragriph v1.2.16.1.^[23]^ Pymol was used for structure analysis and figure representations.^[24]^ OriginLab was used for spectra analysis and figure representation of HPLC-chiral experiment.^[25]^ Sigmaplot 15.0 was used to draw the kinetics figures.

# **Figures**

**Figure S1:** GC/MS chromatogram of the reaction performed with 2-oxobutanoate (**2**) and phenoxy-2-propanone (**6**). The reaction catalyzed by JanthE K567S was used for figure representation. Peak at 8.1 min represents the acceptor substrate (**6**).

**
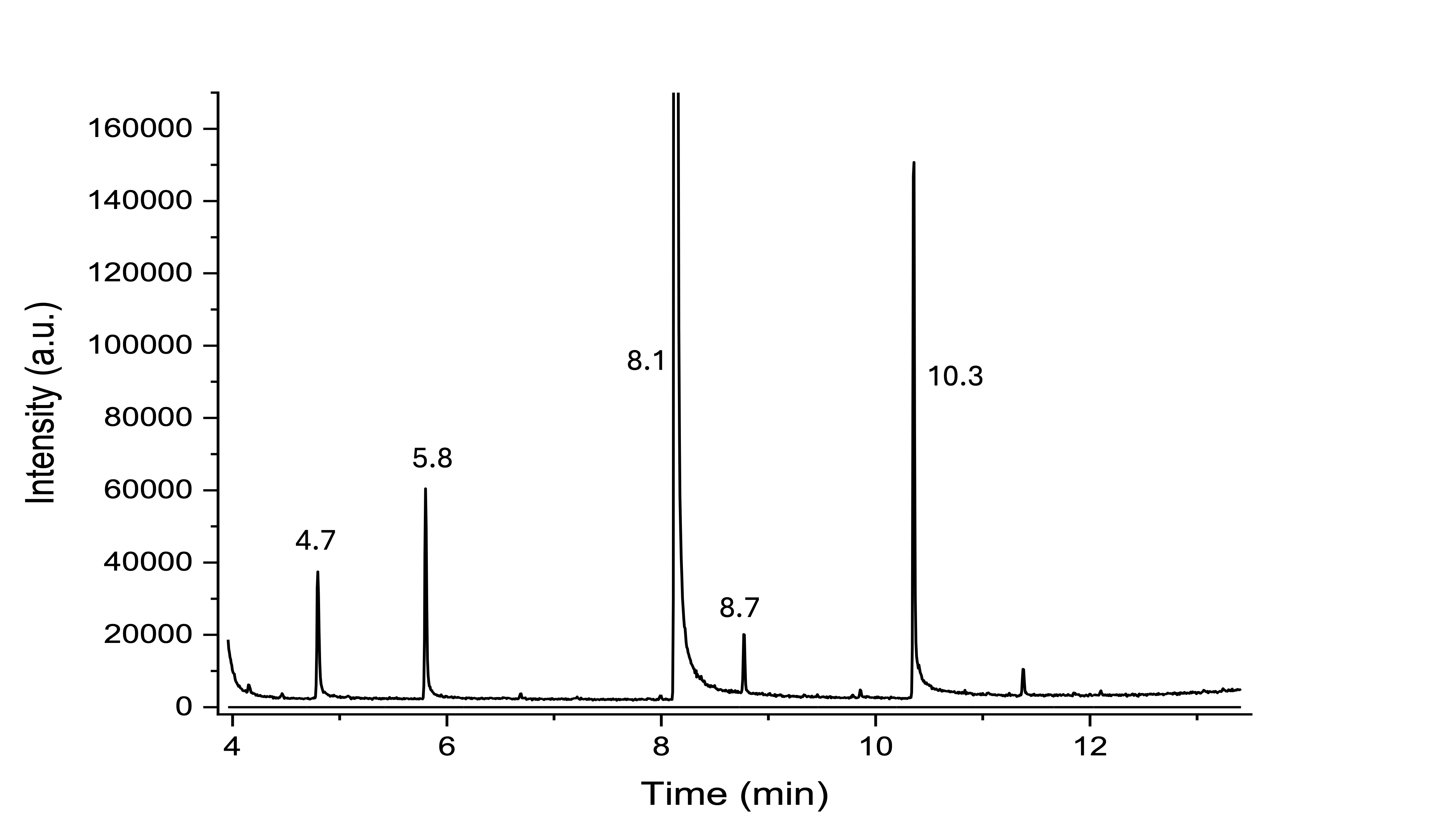
**

**Figure S2**: Mass spectra of product **9** and side products **10**, **11**, and **12** obtained with GC/MS. **
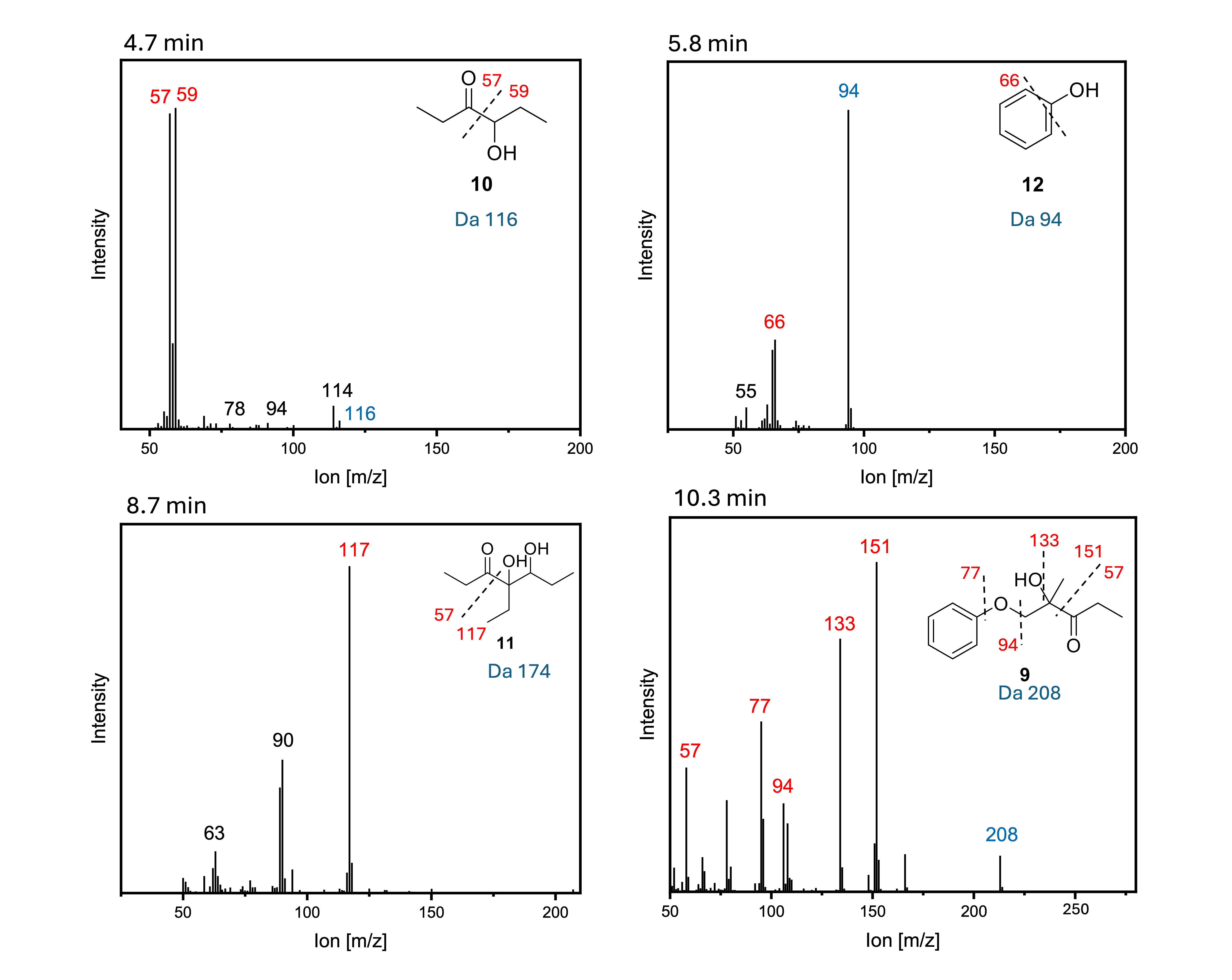
**

**Figure S3:** GC/MS chromatogram of the reaction catalyzed with 2-oxovalerate (**3**) and phenoxy-2-propanone (**6**). The reaction catalyzed by JanthE K567S was used for figure representation. Side product masses are presented in the lower panel. Peaks at 8.1 and 10.8 min represent the acceptor substrate (**6**) and product (**13**).

**
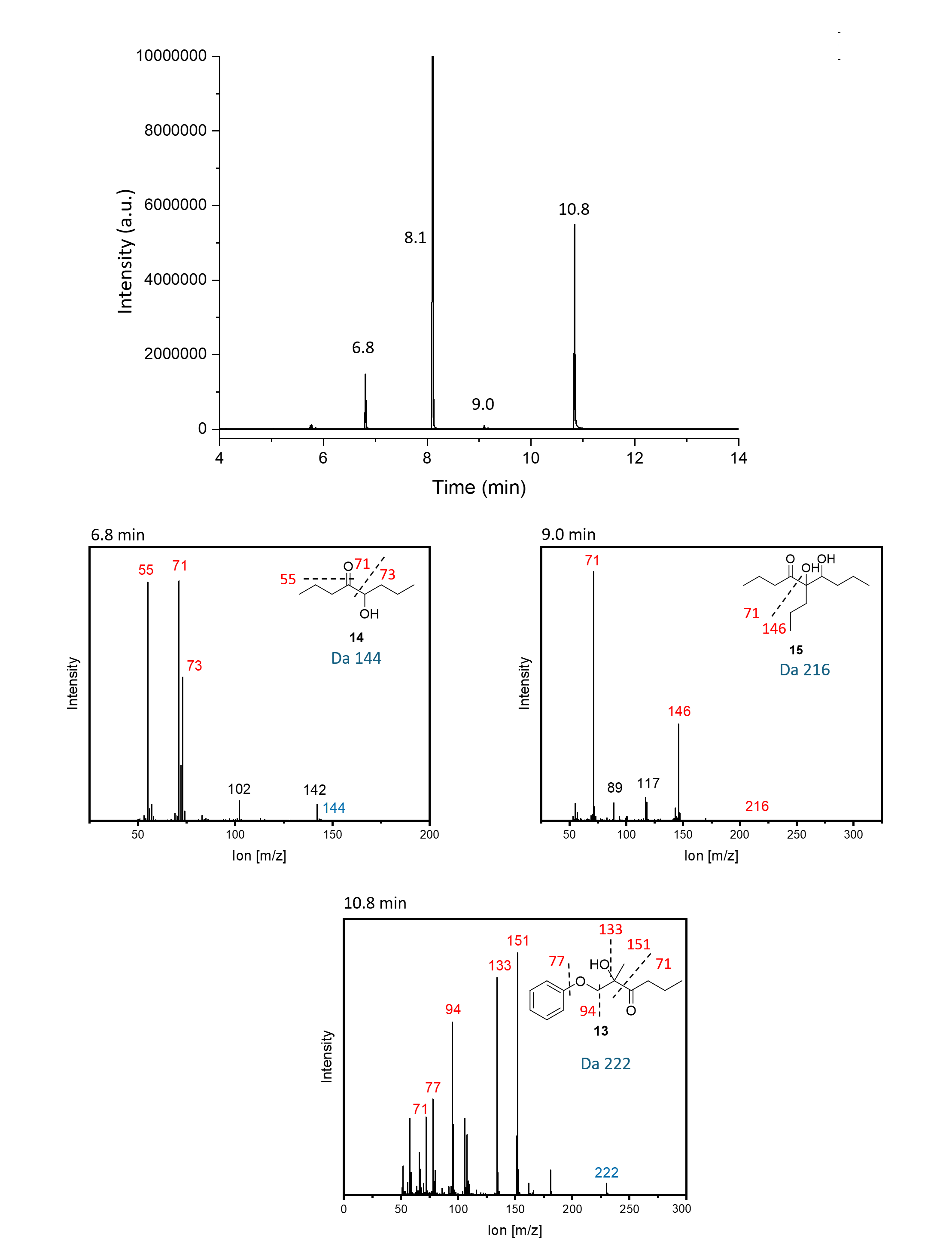
**

**Figure S4:** Other tested variants of JanthE in which conversion to product **9** was lower or comparable to the wild type. Data are representative of a single experiment performed using cell-free extract normalized for pellet weight. Mutating V121A, P293A, Y268A, and Y268H results in a decrease of product **9** formation compared to the wild type, suggesting that these positions are pivotal for enzymatic catalysis. Mutating Y297 to glutamic acid also resulted in lower activity.

**Figure S5:** Comparison of conversions to product **9** obtained with single variant at position K567, Y297, V121, and double variants using purified enzyme. Single mutations at position Y297P and Y297F were found by site saturation mutagenesis. All variants at positions Y297 and the V121I variant do not substantially increase reaction conversions, while V121M results in higher conversions compared to the wild type. The increment is not higher than K567S. Double variants at positions Y297 and K567 do not show better conversions compared to single variants at position K567. Data are representative of an experiment conducted in duplicate in 250 µL assay.


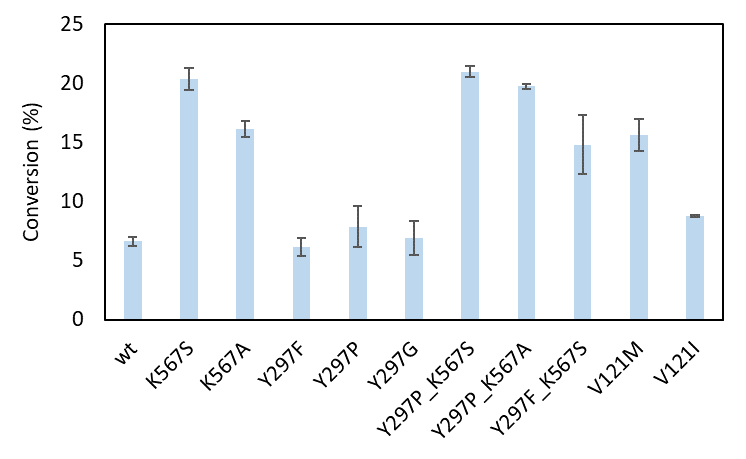


**Figure S6:** Enantiomeric excess of compound **9** produced by wild type JanthE compared to variants.
**A**) UV-vis spectrum of product rac-**9** recorded on chiral-phase HPLC. Maximum absorbance peaks are observed are 225 nm and 270 nm. **B**) Comparison of the raw chromatogram of rac-**9** with the enantioselective synthesis by wild type JanthE and variants K567S, K567A, and K567S_Y297P.
**C**) Comparison of enantioselectivity of wild type JanthE and variants. Data are representative of average of triplicate experiments.


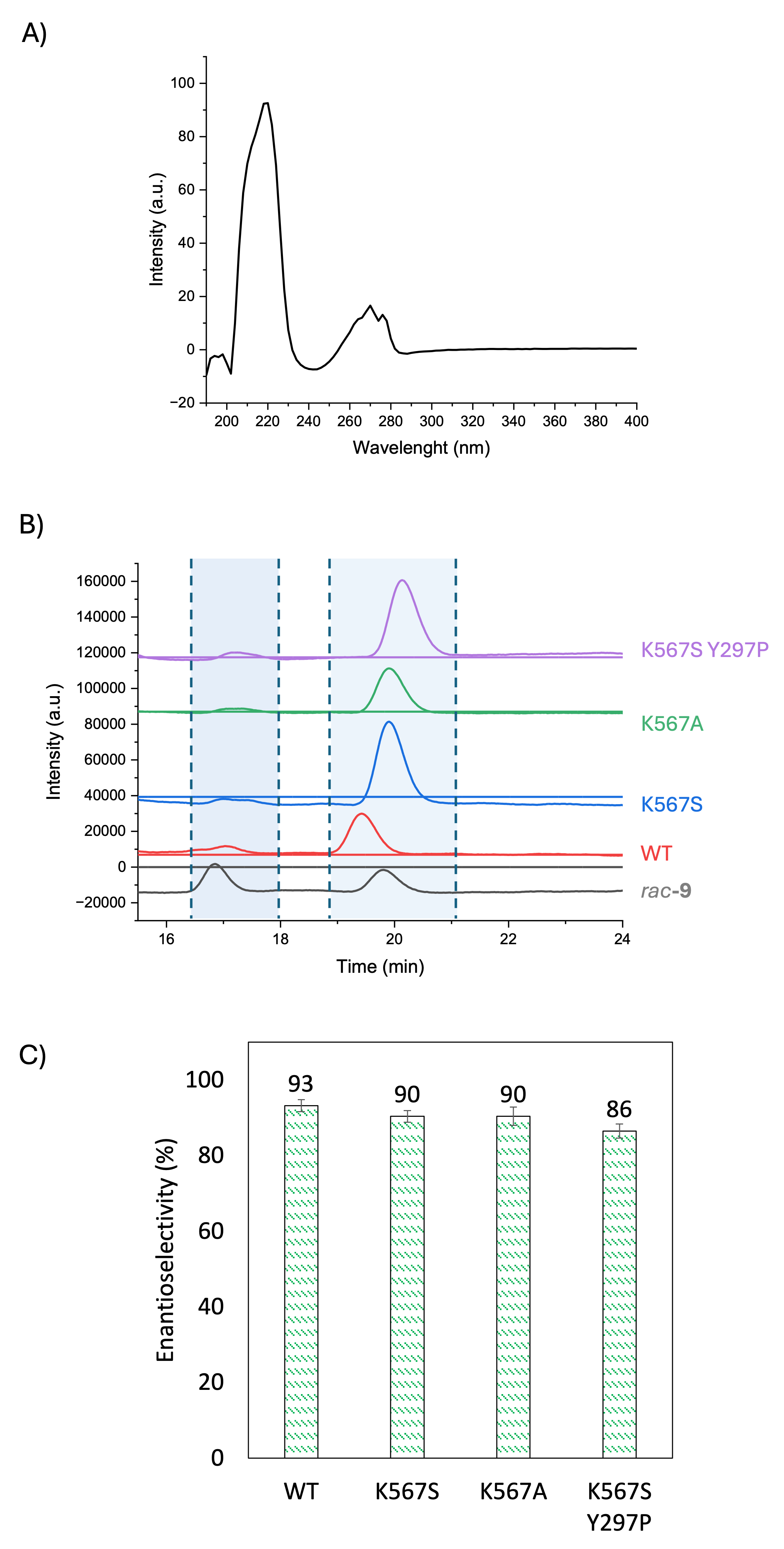


**Figure S7:** CD spectra of (R)-**9** synthesized by JanthE K567S. The spectra were recorded with (**A**) 2.5 µL or (**B**) 0.5 µL of product dissolved in 300 µL acetonitrile. Acetonitrile was used as blank. A negative cotton effect with a maximum at 295 nm is observed.

**Figure S8:** Area of product **9** formed after 4, 24, and 48 h. This experiment was performed in a volume of 250 µL and enzyme concentration of 1 mg·mL^–1^. The area represents raw data. No normalization for extraction has been performed and data is representative of a single experiment. These data serve as a starting point for further optimization of process conditions.


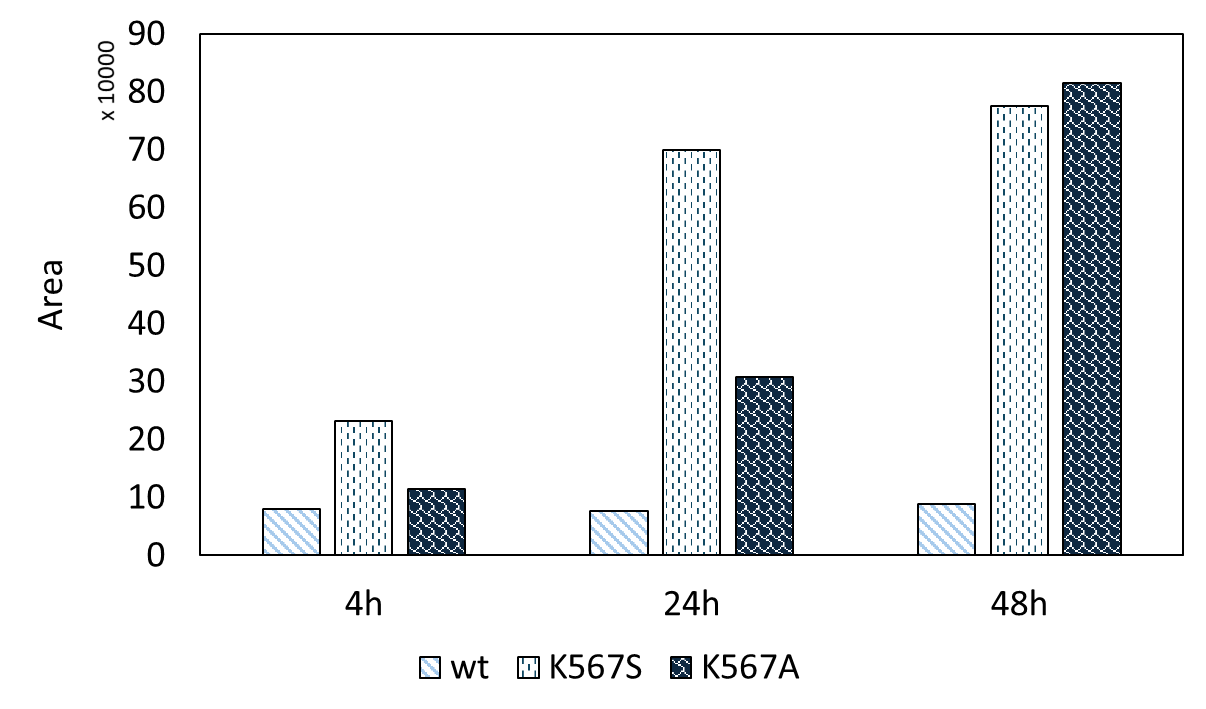


**Figure S9:** Dependence of the specific activity of JanthE wild type (wt) and variant JanthE_K567S (c_Mg2+_ = 1 mM, c_ThDP_ = 0.05 mM, c_JanthE_ = 1 mg·mL^−1^, 100 mM sodium phosphate buffer pH 8, c_NaCl_ = 200 mM) on the concentration of 2-oxobutanoate (c_phenoxy-2-propanone_ = 20 mM) for the formation of the side product **10**. Estimated kinetic parameters of JanthE wild type (wt) and K567S variant are reported in the table. Data are representatives of triplicate experiments.

| Parameter | Unit | wt | K567S |
| --- | --- | --- | --- |
| *V_m_* | U mg^-1^ | 0.008 ± 0.002 | 0.034 ± 0.011 |
| *K_m_*^2-oxobutanoate (^**^2^**^)^ | mM | 5.12 ± 2.63 | 14.68 ± 7.37 |
| *K_i_*^2-oxobutanoate (^**^2^**^)^ | mM | 49.0 ± 27.0 | 41.92 ± 22.77 |

**Figure S10:**  ^1^H NMR and ^13^C NMR spectra of enzymatically synthesized and isolated product **9**. The spectra were recorded with 2.5 mg of product in CDCl_3_.

**
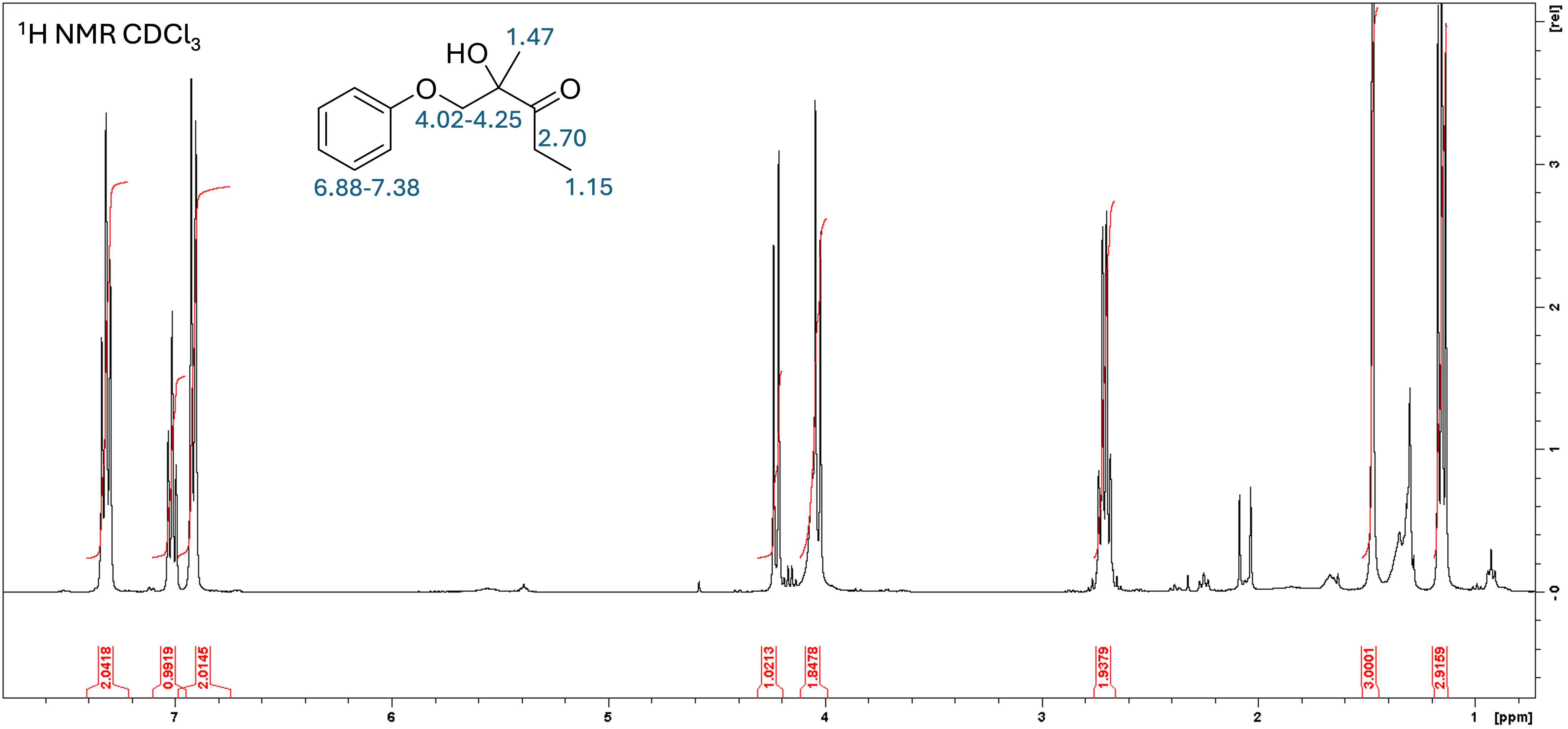
**

**
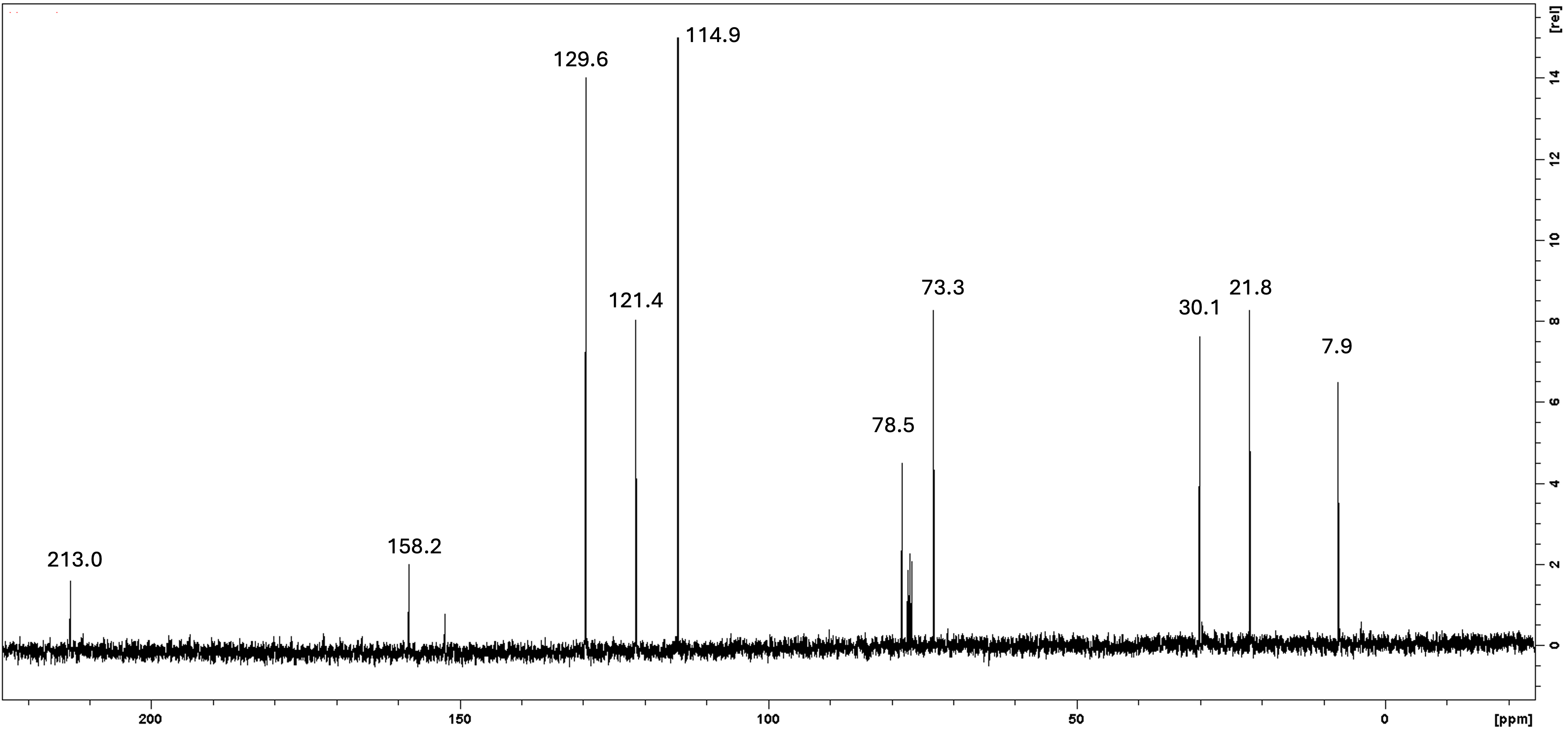
**
